# Long Non-Coding RNA *TUG1* Expression Is Associated with Different Subtypes in Human Breast Cancer

**DOI:** 10.3390/ncrna3040026

**Published:** 2017-12-20

**Authors:** Daniela F. Gradia, Carolina Mathias, Rodrigo Coutinho, Iglenir J. Cavalli, Enilze M. S. F. Ribeiro, Jaqueline C. de Oliveira

**Affiliations:** Department of Genetics, Federal University of Parana, Curitiba 81531980, Brazil; danielagradia@gmail.com (D.F.G.); carol.mathias1@hotmail.com (C.M.); rodrigocout@gmail.com (R.C.); cavalli@ufpr.br (I.J.C.); enilzeribeiro@gmail.com (E.M.S.F.R.); jaqueline.genetica@gmail.com (J.C.O.)

**Keywords:** *TUG1*, TCGA, breast cancer, PAM50, survival, lncRNA

## Abstract

Taurine upregulated 1 gene (*TUG1*) is a long non-coding RNA associated with several types of cancer. Recently, differential expression of *TUG1* was found in cancerous breast tissues and associated with breast cancer malignancy features. Although this is evidence of a potential role in breast cancer, *TUG1* expression could not be associated with different subtypes, possibly due to the small number of samples analyzed. Breast cancer is a heterogeneous disease and, based on molecular signatures, may be classified into different subtypes with prognostic implications. In the present study, we include analysis of *TUG1* expression in 796 invasive breast carcinoma and 105 normal samples of RNA sequencing (RNA-seq) datasets from The Cancer Genome Atlas (TCGA) and describe that *TUG1* expression is increased in HER2-enriched and basal-like subtypes compared to luminal A. Additionally, *TUG1* expression is associated with survival in HER2-enriched patients. These results reinforce the importance of *TUG1* in breast cancer and outline its potential impact on specific subtypes.

## 1. Introduction

Over the past few years, there has been increasing widespread interest in long non-coding RNAs (lncRNAs). LncRNAs are non-protein coding transcripts greater than 200 nucleotides in length and lacking in significant open reading frames [1]. These transcripts with their complex gene architecture have attracted the significant attention of researchers due to their recently discovered role in the development of some human diseases [2] as regulators of transcriptional and post-transcriptional gene expression [3]. It is estimated that the number of lncRNAs in the human genome is in the tens of thousands [4]. In addition, for the majority of these transcripts, their biological roles and molecular functions still remain unknown.

However, for some lncRNAs, their functions have been determined as involving inhibition of microRNAs (miRNAs), epigenetic regulation, chromatin state modification and chromosomal interactions, transport of proteins, and, finally, modulation of the activity or abundance of proteins or messenger RNAs (mRNAs) with which they interact [5].

Taurine upregulated 1 gene (*TUG1*) is a 7.1 kb lncRNA transcribed from human chromosome 22q12.2 [6]. Accumulating evidence shows that *TUG1* plays an important role in several types of cancer [7].

Recently, Li et al. [8] detected hyperexpression of *TUG1* in 100 samples of cancerous breast tissue and associated it to breast cancer malignancy features such as tumor size and distant metastasis. Additionally, *TUG1* was associated to Ki-67 and human epidermal growth factor receptor 2 (ERBB2/HER2). Although this study evidenced the potential role of *TUG1* in breast cancer, they failed to examine *TUG1* expression in different subtypes, possibly due to the small number of samples analyzed [8].

Breast cancer is a heterogeneous disease at both the morphological and molecular level. Based on molecular signature Prediction Analysis on Microarrays that uses a minimal gene set (PAM50), there are five main subtypes of breast cancer: basal-like, HER2-enriched, luminal A, luminal B, and normal-like. These subtypes have specific biological features, epidemiology, and prognostic implications, and are useful in guiding patient treatment [9,10].

In the present study, we include analysis of *TUG1* expression in 796 invasive breast carcinoma samples and 105 normal samples of RNA sequencing (RNA-seq) datasets from The Cancer Genome Atlas (TCGA), and describe that *TUG1* expression is increased in HER2-enriched and basal-like subtypes compared to luminal A. Additionally, *TUG1* expression is associated with overall survival in HER2-enriched patients. These results reinforce the importance of *TUG1* in breast cancer and evidence its potential impact on specific subtypes.

## 2. Results

### 2.1. *TUG1* Expression Is Increased in HER2-Enriched and Basal-Like Compared to Luminal A 

The transcript level of *TUG1* in breast cancer tissues was not differentially expressed when compared to adjacent non-cancerous tissue when all samples were considered (*p* = 0.18) nor in paired comparison analysis (*p* = 0.12).

**Figure 1:**
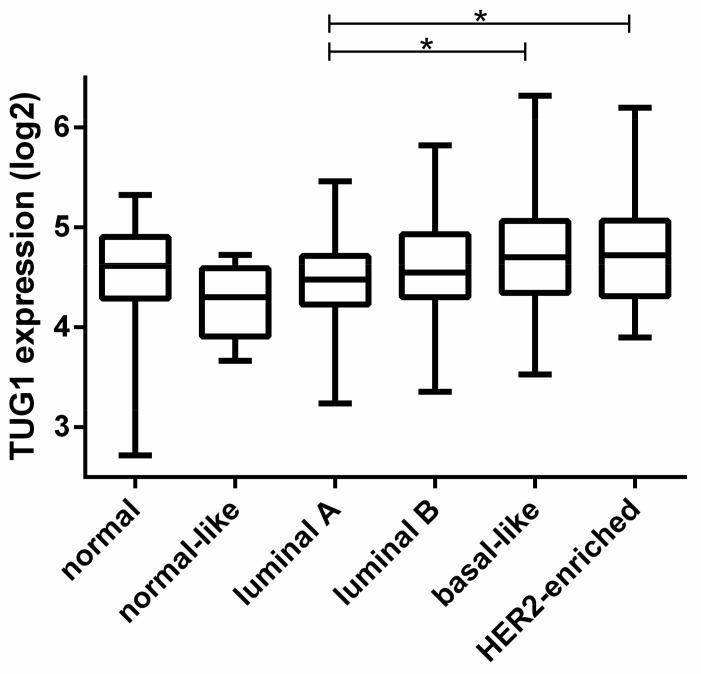
Expression analysis of the taurine upregulated 1 gene (*TUG1*) transcript in Prediction Analysis on Microarrays that uses a minimal gene set (PAM50) breast cancer subgroups. *TUG1* expression data were from “The Cancer Genome Atlas (TCGA)”, and the PAM50 gene signatures rely on 50 discriminatory genes to segregate tumors into luminal A (*n* = 227), luminal B (*n* = 122), human epidermal growth factor receptor 2 (HER2)-enriched (*n* = 56), basal-like (*n* = 94), and “normal-like” (*n* = 7). Samples categorized as “normal” are adjacent non-tumor breast tissue (*n* = 105). * *p* < 0.001.

**Figure A1:**
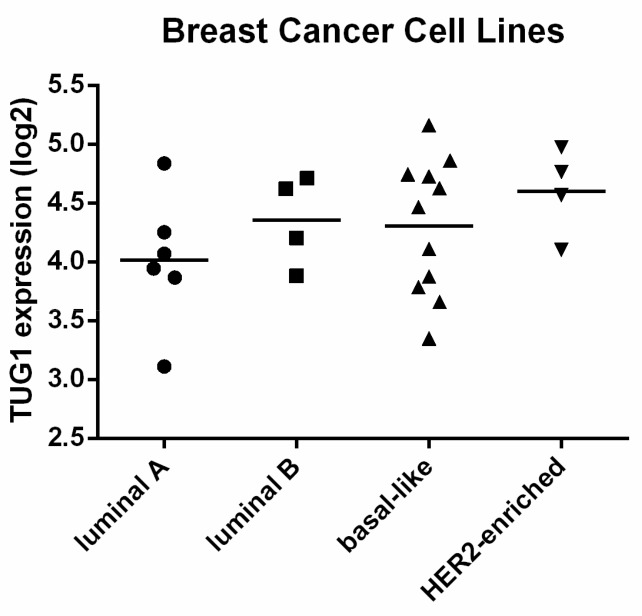
Expression analysis of *TUG1* transcript in cell lines. *TUG1* expression data were from “TCGA. Symbols represents the number of cell lines analyzed: luminal A (*n* = 6), luminal B (*n* = 4), HER2-enriched (*n* = 4) or basal (*n* = 11). Analysis of variance ANOVA was used to examine *TUG1* expression across cell lines (*p* = 0.39).

Related to subtype PAM50 classification, *TUG1* was found to be hypoexpressed in patients classified as having the luminal A subtype when compared to HER2-enriched and basal-like subtypes (*p* < 0.001) (Figure 1). In breast cancer cell lines, although *TUG1* expression profile were similar to that seen in patients, the difference was not significant (*p* = 0.39) (Figure A1).

**Figure 2:**
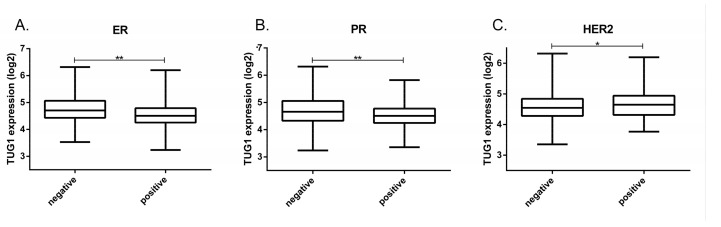
*TUG1* transcript expression associated with breast cancer relevant receptors; (**A**) *TUG1* expression in positive (*n* = 385) or negative (*n* = 114) estrogen receptor (ER); (**B**) *TUG1* expression in positive (*n* = 328) or negative (*n* = 170) progesterone receptor (PR); (**C**) *TUG1* expression in positive (*n* = 74) or negative (*n* = 413) HER2. *TUG1* expression data were from TCGA. * *p* = 0.001; ** *p* < 0.0001. ER, estrogen receptor; PR, progesterone receptor.

High *TUG1* expression was also associated with positive HER2 and negative status for estrogen and progesterone receptors (ER/PR) (*p* ≤ 0.001) (Figure 2). Association with the neoplasm disease stage was not found (*p* = 0.43) (data available at TANRIC platform)

### 2.2. *TUG1* Has a Different Impact on Survival Depending on Molecular Subtype

**Figure 3:**
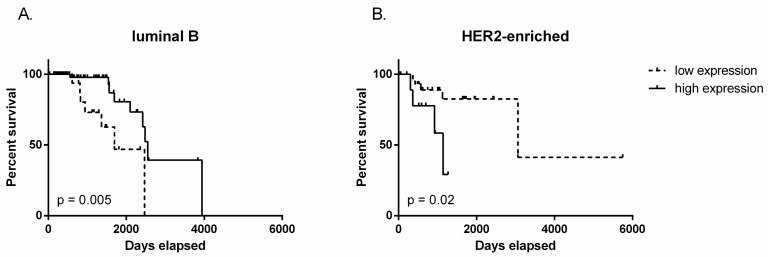
Disease-free survival determined by Kaplan-Meier plots and the log-rank test according to *TUG1* expression. (**A**) luminal B: 25% of patients with lower and 75% of patients with higher *TUG1* expression; (**B**) HER2-enriched: 75% of patients with lower and 25% of patients with higher *TUG1* expression. *TUG1* disease-free survival information of breast cancer patients was downloaded from the TCGA portal.

Considering the differences in TUG1 in molecular subgroups, we investigated the prognostic significance of expression in each subtype. We found that decreased expression of TUG1 is associated with improved disease-free survival in HER2-enriched patients (the lowest 75% expression -P75, *p* = 0.02) but high expression of TUG1 is associated with increased disease-free survival in luminal B patients (the lowest 25% expression - P25, *p* = 0.005) (Figure 3), suggesting that the prognostic value of TUG1 could be different in specific subtypes.

The classification of mammary tumors by the TNM system is based on the main morphological attributes of malignant tumors, which are clinically and histopathologically determined. The parameters evaluated are: primary tumor extension (T), presence and extent of regional lymph node metastases (N), and the presence of distant metastases (M) [11,12]. For subtypes evidenced in univariate analysis of survival, multivariate Cox regression was performed. Age at initial pathologic diagnosis, tumor size (T1 to T4), involvement of lymph nodes (N0 to N3), and presence of metastatic disease (M0 or M1) were considered as covariates, along with *TUG1* expression. In luminal B samples (Table 1), all the covariates tested failed to show significance. However, in HER2-enriched samples (Table 2), the analysis concluded that *TUG1* expression, age at initial pathologic diagnosis, and positive metastasis are associated with prognosis.

## 3. Discussion

The lncRNA *TUG1* was initially identified in developing murine retinal cells, upregulated by taurine, and necessary for the proper formation of photoreceptors [6]. Associated with chromatin-modifying complexes, *TUG1* plays a role in gene regulation, including repression of genes involved in cell cycle regulation [13], and it has been extensively associated with diverse tumor types.

For example, *TUG1* is highly expressed and associated with poor prognostic markers in bladder, hepatic, colorectal, gastric, renal, and ovarian cancer. Additionally, *TUG1* silencing inhibits proliferation and induces apoptosis in cells derived from bladder cancer, osteosarcoma, hepatic, renal, colon, and gastric cancer; it is also associated with migration, invasion, tumorigenicity, the cell cycle, and angiogenesis in many other tumor types [7,14,15,16]. Although most of these works presented *TUG1* as having an oncogenetic role, it is downregulated and associated with better survival in glioblastoma and non-small cell lung cancer, and *TUG1* may function as a tumor suppressor in these cancer cell models [17,18]. Recent papers have reviewed *TUG1* expression in different kinds of cancer [19,20]. Through meta-analysis studies, they investigated the relationship of *TUG1* with clinicopathological features in cancer to delineate the potential clinical applications of this lncRNA on prognosis. Although some conflicting results were found, both studies indicated that increased lncRNA *TUG1* is an independent prognostic biomarker for unfavorable overall survival in cancer patients.

The involvement of *TUG1* in breast cancer is still controversial. High expression of *TUG1* was found in 100 cancerous tissues and associated with poor features such as tumor size, distant metastasis, and TNM (Tumor, Node, Metastasis) staging [8]. High expression was also observed in 24 breast cancer samples when compared to adjacent tissues and potential oncogenic functioning of *TUG1* in breast cancer cell lines was evidenced by retardation of cell proliferation, cell migration, and invasion; suppressed cell cycle progression; and increased apoptosis after knockdown of *TUG1*. By another side, Fan and colleagues [21], studying 58 matched pairs of breast cancer and normal tissue samples, observed that *TUG1* overexpression promoted cell migration and invasion while *TUG1* knockdown had the opposite effects, suggesting that *TUG1* is a tumor suppressor in breast cancer.

Technological advances are generating enormous amount of data, and the public accessibility of this information is accelerating the connection between scientific research and clinical application. Cancer genome data from large-scale projects such as TCGA is being used to discover new relevant aspects of cancer genomics such as the development of therapeutic strategies and identification of specific biomarkers.

We included expression analysis of *TUG1* in 796 tumor samples’ and 105 normal breast tissue samples’ RNA-seq datasets from TCGA. This large number of samples enables broad analyses, allowing, for example, the possibility of focusing on the specific nuances of each subtype.

High expression of *TUG1* in breast cancer tissues compared to adjacent tissues was not confirmed in the present study; herein *TUG1* expression was quite similar in tumor and control samples.

Li et al. [8] found *TUG1* was upregulated in invasive breast cancer, more specifically in Ki67 and HER2-positive patients, however, they failed to examine differences between subtypes, a limitation of their paper. The present study, based on TCGA analysis, described high *TUG1* expression in basal-like and HER-enriched patients compared to luminal A patients. Additionally, high expression of *TUG1* was confirmed in HER2-positive patients, and we also found high expression in ER- and PR-negative patients.

PAM50 classification provided some additional biological insights into molecular subtypes. These subtype groupings have important implications in clinical practice, mainly for the identification of patients for whom chemotherapy could be avoided, such as low risk endocrine-positive patients, usually HER2-negative [10]. Furthermore, there are many molecular mechanisms to be better understood in these subtypes considering, for example, identification of novel therapeutic targets, mainly in high-risk patient groups in which chemotherapy has only modest benefits.

High expression of *TUG1* was found in HER2-enriched and basal-like patients in comparison to luminal A, usually low risk, endocrine-positive patients. HER2-enriched and basal-like patients have a higher risk of relapse and may not be susceptible to the effects of anti-HER2 target treatment (mainly in the basal-like group) [22]. *TUG1* expression in these subtypes may be acting in the molecular pathways but additional investigation is needed to understand the specific networks.

Survival analysis considering all patients with breast cancer did not evidence differences but we found correlations between *TUG1* expression and overall-survival in patients classified as luminal B or HER2-enriched: patients with the lowest *TUG1* expression had lower survival in luminal B patients, while the highest *TUG1* expression was associated with poor overall survival in HER2-enriched patients.

In multivariate Cox regression including *TUG1* expression and other clinical factors (age at initial pathologic diagnosis and TNM classification), the results from the luminal B samples failed to demonstrate an association. There were a great number of censored samples at the beginning of survival analysis, so these results should be viewed with caution. The multivariate results from HER2-enriched samples showed that *TUG1* expression, age at initial pathologic diagnosis, and distant metastasis were significantly correlated with prognosis, however, it is important to take into account that the number HER2-enriched samples presenting with distant metastasis (*n* = 2) was too small to draw any reliable conclusions.

*TUG1* mechanisms of action and regulation is not completely known. Like its complex clinical associations, its mechanism of cellular action appears to be diverse.

The first mechanism described shows that *TUG1* is induced in p53-wild type cells, but not in p53-mutant cells, and binds to polycomb repressive complex 2 (PRC2) to silence genes involved in cell-cycle regulation [13].

*TUG1* was found to decrease miR-145 expression; more specifically, there was a reciprocal repression between *TUG1* and miR-145. This regulation may induce expression of many miR-145 targets, for example, the zinc finger E-box binding homeobox 2 (ZEB2) [23]. Additionally, *TUG1* may be induced by nuclear transcription factor SP1 and repress, by polycomb repressive complex 2 (PRC2), Kruppel-like factor 2 (KLF2) transcription [16].

Notch1 activation may induce *TUG1* expression [24], and this lncRNA possibly interacts with Wnt/β-catenin signaling. On the other hand, the potential tumor suppression function of *TUG1* may be considered, for example, by its regulation of PTEN expression [25].

As well as *TUG1* mechanisms seeming to be complex and associated with multiple signaling pathways, its clinical relevance seems to depend on the context of each subtype.

Additional studies are needed to better understand how *TUG1* is included in molecular pathways in each subtype. The recognition that lncRNAs such as *TUG1* are differentially expressed in different subtypes, and recognition of their relationships to known molecular pathways for a subtype, opens important perspectives toward this aim.

In conclusion, *TUG1* expression was found to be higher in patients classified as HER2-enriched and basal-like, compared to luminal A. Additionally, *TUG1* expression was associated with survival in HER2-enriched patients. These findings suggest a potential role of this lncRNA in specific subtypes of breast cancer and in the development of different phenotypes. Further studies are needed to corroborate and extend our results.

## 4. Patients and Methods 

Information from 796 invasive breast carcinoma and 105 non-neoplastic cancer-adjacent breast tissues was collected. All RNA-seq data and clinical information is available in the TCGA database following ethics, laws, and policies from the program. Clinicopathological characteristics of the studied population is presented in Table 3.

Clinical guidelines for determining estrogen receptor (ER), progesterone receptor (PR), and human epidermal growth factor receptor 2 (ERBB2/HER2) status for breast cancers have been established in TCGA samples. According to the current clinical guidelines jointly issued by the American Society of Clinical Oncology (ASCO) and the College of American Pathology (CAP), in use since January 2010, a breast tumor is considered ER- and PR-positive if the immunohistochemistry (IHC) value of the corresponding nuclear staining is ≥1%. Before 2010 there was no universal standard for ER and PR status, so local hospitals used their own thresholds for their clinical practices. Considering that the year of diagnosis of all breast cancer cases collected in this study ranged from 1988 to 2011, determining the clinical status for ER and PR followed a mixture of thresholds. For HER2, following the current ASCO/CAP guideline, a breast tumor with IHC values of 0 or 1+ is called “Negative”, level 2+ is “Equivocal”, and level 3+ is “Positive”. The equivocal cases are then analyzed by Fluorescence In Situ Hybridization (FISH), where a case is called “Positive” if the FISH ratio is ≥2.2, and “Negative” if the FISH ratio is ≤1.8 [26]. Subtype stratification was based on the identification of gene expression by the 50-gene PAM50 predictor [27].

Large-scale RNA-seq datasets from The Cancer Genome Atlas (TCGA) were assessed and analyzed by the open-access web resource “The Atlas of Noncoding RNAs in Cancer” (TANRIC) [28]

On the TANRIC website, RNA-seq datasets for breast cancer cell lines are also available. We included *TUG1* expression information from 25 cell lines, classified as luminal A (*n* = 6), luminal B (*n* = 4), HER2-enriched (*n* = 4), or basal (*n* = 11) subtypes [29,30].

Analysis of variance (ANOVA) with post-hoc Tukey testing was used to examine *TUG1* expression across tumor subtypes and disease stages. Student’s *t*-test assessed statistical differences between positive and negative ER, PR, and HER2 status. The homogeneity among the variances was tested by Bartlett’s test. More details of the algorithm for expression quantification and statistics are better described by Li et al. [28].

For survival analysis, patients were stratified into values below and above the median and the quartiles (P25 and P75). The Kaplan-Meier curves and log rank tests were used to estimate overall survival, which was calculated to the last follow-up, or death event. Additionally, multivariate Cox proportional hazards regression analysis included *TUG1* expression, age, tumor size, involvement of lymph nodes, and presence of metastatic disease, with calculation of the hazard ratio (HR) and 95% confidence interval (95% CI).

Data were analysed statistically using the Statistical Package for the Social Sciences (SPSS) software for Windows, version 15.0 (SPSS Inc., Chicago, IL, USA). Cox proportional hazards regression analysis was performed using the R package [31].

## Figures and Tables

**Table 1:** Multivariate Cox analysis of luminal B samples.

Multivariate Cox Analysis		
Covariate	Hazard Ratio (95% CI)	*p*-Value
Age at initial diagnosis	1.0038 (0.9480–1.063)	0.897
*TUG1* Expression	0.5427 (0.1539–1.914)	0.342
Tumor Size (T1, *n* = 17; T2, *n* = 84; T3, *n* = 15; T4, *n* = 6)	1.4296 (0.4925–4.150)	0.511
Node Metastasis (N0, *n* = 52; N1, *n* = 46; N2, *n* = 16; N3, *n* = 8)	0.8022 (0.2955–2.178)	0.665
Distant Metastasis (M0, *n* = 114; M1, *n* = 5)	3.3331 (0.2284–48.651)	0.379

CI, confidence interval. T1–T4, tumor size; N0–N3, involvement of lymph nodes; M0–M1, absence/presence of metastatic disease

**Table 2:** Multivariate Cox analysis of HER2-enriched samples.

Multivariate Cox Analysis		
Covariate	Hazard Ratio (95% CI)	*p*-Value
Age at initial diagnosis	1.130 (1.0110–1.264)	**0.0314**
*TUG1* Expression	37.262 (2.5634–541.647)	**0.00807**
Tumor Size (T1, *n* = 8; T2, *n* = 36; T3, *n* = 8; T4, *n* = 3)	2.374 (0.5688–9.904)	0.23564
Node Metastasis (N0, *n* = 21; N1, *n* = 17; N2, *n* = 11; N3, *n* = 6)	1.757 (0.6721–4.593)	0.25039
Distant Metastasis (M0, *n* = 53; M1, *n* = 2)	27.257 (1.2025–617.812)	**0.03791**

CI, confidence interval. T1–T4, tumor size; N0–N3, involvement of lymph nodes; M0–M1, absence/presence of metastatic disease. Bold entries denote statistically significant values.

**Table 3:** Clinicopatholigical Features.

Characteristics		
Gender	Male (*n* = 8)	Female (*n* = 784)
Age at diagnosis	≤35 (*n* = 27)	>35 (*n* = 765)
ER status	Negative (*n* = 173)	Positive (*n* = 582)
PR status	Negative (*n* = 247)	Positive (*n* = 506)
HER2 status	Negative (*n* = 628)	Positive (*n* = 113)
Tumor Size	≤2cm (*n* = 202)	≥2cm (*n* = 560)
Node Metastasis	Negative (*n* = 369)	Positive (*n* = 396)
Distant Metastasis	Negative (*n* = 745)	Positive (*n* = 14)

Patient information from TCGA. Data only for those with *TUG1* expression and clinical data available (*n* = 796).
